# On Medical Education

**Published:** 1828-04-01

**Authors:** 


### MEDICAL EDUCATION.

In approaching this important subject, once more, we feel that, however unequal we may be to the task, we shall bring to the investigation a freedom from prejudice, and complete independence of mind. We shall endeavour to discard from our thoughts (as far as possible) the impressions made by the writings of others, in order that we may examine for ourselves, and draw those deductions that should naturally follow an unbiassed and unimpassioned inquiry. The two great points of examination, in our opinion, are, *first*, what is the actual state of medical education in this country, and what are its defects?—*secondly*, what are the means most likely to remedy these defects, if they are found to exist?

We will suppose that a sovereign or a subject of this realm (for, on the bed of sickness, there is a sad levelling of ranks) is stricken down with any formidable disease—say gout in the stomach, and inflammation in the foot. The best aid that the healing art can afford is called in, and we will suppose Sir Henry Halford, Sir Astley Cooper—and Mr. Teggart, in consultation on the case. These gentlemen investigate the disease, and determine on the remedies. It would be consonant with reason and common sense that, were these gentlemen all educated and trained to the study of medical science in the best possible way, so they would bring to the treatment of the disease the greatest amount of professional information. But these three gentlemen, who may represent the three great institutions presiding over the profession, are educated in three different ways—and grow up with three very different views of the basis of medical knowledge. These three ways or views cannot all be the best—it is more than probable that not one of them is the best that could be devised. The representative of the College of Physicians is taught to consider a long and expensive study of Greek, Latin, Mathematics, and Philosophy, in two specific towns of England, as the sine qua non—the beau ideal of medical science—and laws are framed accordingly. The representative of the College of Surgeons has been trained in a very different way of thinking. His institution looks to anatomy, physiology, surgery, and the clinical practice of hospitals, as the sure path to the summit of medical knowledge. But this institution, too, has its peculiar views (if not prejudices) of study. Anatomy can only be learnt when the sun is in six particular signs (out of twelve) of the

Zodiac. The anatomy acquired under ARIES is good that under SCORPIO, is—good for nothing! The *point* which the mercury indicates in the thermometer, is, according to this Institution, the *grand point* in anatomy. Neurology, for example, if studied with the mercury at 32° would be excellent—but if studied at 65°, would be useless to the dissector! The acquirement of minute anatomy, according to this presidency, is nothing, when compared with the particular street or town in which it is acquired. The smallest *quantum* of anatomical knowledge is *cognizable* in the one case—but the largest amount is perfectly *incognizable* by collegiate optics in the other! The same rules are applicable to the elementary lectures on surgery. In respect to CLINICAL study, it was reserved for the moderns to discover that the number of square feet, which the wards of an hospital cover, is of infinitely more importance than the ability of the medical officers—the pains which they take to explain diseases—or the assiduity of the students. It has been ascertained, indeed, that a great analogy exists between public instruction and private practice. The number of patients which a surgeon visits in the day, makes the great or the little surgeon. The number of beds which a student runs past in an hospital, makes the eligible or the ineligible candidate for collegiate honours! Sound surgery, in fact, like lead or tin in the mines of Cornwall, has been ascertained to run in *veins*—and the collegiate miners have an intuitive knowledge of the direction of these veins, without any trouble of boring and digging into the soils which contain them! They have determined that there are certain meridian lines or parallels, along which the ore of surgical science can *only* run—and, consequently, that it would be labour lost to seek it in any other channel.*

But the representative of the College of Apothecaries entertains (from education and habits) a notion extremely dissimilar to either of the above. His creed is, that a knowledge of the external characters and pharmaceutical affinities of certain simples and compounds, is of far greater importance to the medical student, than a knowledge of the complicated structure and functions of the human frame to which they are applied. According to him and his College, five years of hard labour behind the counter, are essentially necessary to learn the noble art and mystery of rolling pills, filtering tinctures, mixing draughts, writing labels—and, what is of still more importance, posting the ledger—while 18 months or two years, are quite enough for the acquirement of that knowledge which consists in examining the structure of the human body—studying its natural and disordered functions—becoming familiar with the changes of texture induced by disease—and ascertaining the effects of the oceans of physic which he had dispensed during a long apprenticeship!

Now, in the name of science and common sense, how is it possible that three men coming into consultation, with such different ideas resulting from different modes of education and habits of thought, can amalgamate in their opinions—anatomical, pathological, or therapeutical? It is utterly impossible that any unison of sentiment can prevail in such a conclave—and hence one great source of the discordant ideas among medical men respecting the most common or familiar complaint.

This medical TRINITY, then, has done any thing but conduce to medical

---

* It is true, that these ideas being found to meet with universal disapprobation, have been recently modified—and the liberal party in the Chirurgical Cabinet has had partial success. But the promulgation and subsequent retractation offer the most incontestible evidence of the spirit which governs corporate bodies, when not controlled by the voice of the public, and the animadversions of the press.

UNITY. The existence of three chartered bodies, each having a distinct system of laws for medical education, is such a monstrous anomaly in the *study* of a science, whose principles are the same all over the world, that future generations will hardly credit the fact! It is to this triple-headed monster that the distractions of medical society must mainly be traced. But it may be said that there is a natural propensity in medical men to betake themselves to different branches of the profession. No wonder there should be, when the three great presiding institutions instil such divisions into the minds of youth, from their fourteenth year upwards! But the divisions in *practice* would be of no disadvantage, if there was a unity in the *study* of medicine. The different systems of study not only introduce heterogeneous systems of practice, but they deteriorate the practice of medicine generally. Thus, the line of study prescribed by the College of Physicians disqualifies its members for the practice of *surgery*—the code adopted in Lincoln's-Inn Fields is totally defective, as regards the practice of *physic*—while the system of the Apothecaries' College renders its candidate unfit for *both*, although *he* must practise all branches of the profession nolens volens! It is, we conceive, impossible for any man, not blinded by prejudice or seduced by party, to contemplate the anarchy, the confusion, and the discontent, which now reign in the medical world, without clearly tracing their origin to the distracted councils which promulgate so many different, and so many absurd systems of medical education.

As each of the three chartered bodies has a patent from government for the exclusive sale of a particular pattern of parchment, at an *ad valorem* profit of five thousand per cent, it requires but a very moderate knowledge of human nature, to foresee that the ingenuity of these bodies will be incessantly at work to turn this monopoly of parchment to the advantage of the rulers in the said bodies corporate. The edicts which have issued, and continue to issue, from the Colleges of London and Dublin, too plainly prove the truth of this position. With honey on their lips, these edicts breathe nothing but aspirations for the PUBLIC GOOD; while an analysis of their operation very generally traces their origin to PRIVATE AGGRANDIZEMENT. There cannot be a more dangerous principle infused into the constitution of a corporate body than that which sets LUCRE against VIRTUE—or, in other words, the interest of ONE'S SELF against the interest of ANOTHER. How generally, or rather how universally the *latter* kicks the balance when weighed with the *former*, need not be told in the year 1828.

Now let us inquire whether the corporate bodies above-mentioned, are tinctured with the principle in question? The College of Physicians, like the other two Colleges, draws its principal revenue from the sale of the patent sheep-skins already described. This, in itself, is a very bad principle. It is a principle which must incessantly prompt to extend the amount of sale, without any reference to the qualifications of the purchasers. The College must ardently wish that every physician in Great Britain—nay every physician in the world, might come to its shop, and purchase the licence. In favour of this, SELF-INTEREST continually doth cry! In favour of conferring the diploma *only* on men of eminent science, literature, and talent, to the great defalcation of revenue, the still small voice of *philanthropy*—or the *public good*, is opposed. To which party the College might be inclined to lend a gracious ear, we leave to our readers to guess! The sum of 57 pounds obtained from a country physician, is of some consequence to the College—but what further benefit can the College derive from the issue of the diploma, whether the bearer be considered in his native town, superior to a Baillie—or only a plodding every day physician, without any reputation at all? In such cases, if the interest of SELF

predominates over the interest of ANOTHER, it must be the policy of the College to throw every *facility* in the way of the Licentiate—and remove every thing in the shape of a rigid examination—and especially a public examination, from the sale of the patent parchment.

The very same train of argument applies to the College of Surgeons. It must be the object of that Institution, to place on its list every general practitioner in England. The revenue from the sale of parchment is immense—and, to throw such an impediment as a rigid and public examination in the way of that sale, would be the same as for a shop-keeper to put up a large board before his house, directing his customers to the next door! The College knows its own interest much better. It is fully capable of appreciating the relative value of NUMMUS and VIRTUS—and is far too classical not to adopt the motto of the Roman poet—" VIRTUS *post* NUMMOS."* Independent of the diplomatic revenue, with all its temptations, there is another danger, arising from what may be called " the chapter of accidents.' Thus, where the curriculum of medical studies is left to the decision of a particular chartered body, it is possible that, with the very best intentions, a path might happen to be chalked out for the student, which would necessarily lead him to frequent certain emporia of surgical science, in which the framers of the said curriculum had a deep interest—by which *accident*, other and very excellent emporia might suffer, or be absolutely annihilated! Now this could hardly happen, if the curriculum was framed by a Parliamentary Committee. At all events, the chapter of accidents would be as likely to distribute its favour in one direction as in another.

Let us next look at the legal operations of these three corporate bodies. The College of Physicians has *only* jurisdiction over London and seven miles around—and that *only* over men who sign their names, as they write their prescriptions, in abbreviated characters. But what is worse, the statutes of the College, as now worked, too often operate in the encouragement of ignorance and the repression of knowledge! If a man, without any medical science, sets up for a physician, and even placards his name, as Dr. A. or Dr. B. through the streets, he may destroy as many lives as he can, and no molestation will be offered by the College; but if a man spend ten years in the best universities or medical schools of Europe, and then take out a diploma, after a severe public examination, he will be prosecuted by the College for prescribing in London—unless he pays 57 pounds to the corporation! This proves that it is the price of the licence, rather than the ability of the candidate, which is contemplated by the working of the statute—in other words, that it is VIRTUS *post* NUMMOS.

As to the College of Surgeons, it has no legal power whatever to control unqualified or unprincipled practitioners—and, if newspapers speak truth, it enrols on its lists some of the most unblushing Charlatans of this great metropolis! The concourse of candidates for the diploma of the College is, there-

* We are quite aware (and we have before stated the fact) that in the Council of the College, as in the cabinet of state, there are two parties—the liberals and the illiberals. The latter have been obliged, of late, to relax a little—partly in consequence of the animadversions of the press—partly, from the remonstrances of the liberals. Thus, certificates of medical, chemical, and obstetrical lectures are enjoined in the recent curriculum—but, as no examinations take place on these important subjects, the law is nearly inoperative. Few students will give themselves much trouble in attending to medical and obstetrical studies, when they know that the production of the certificate is all that is required, and that no questions will be asked in medicine or midwifery. This recent promulgation offers another proof of the necessity for a regular curriculum and a public examination, in all the three departments.

fore, produced by a voluntary impulse on the part of surgical practitioners themselves, in order that they may possess the *best* surgical testimony which the medical jurisprudence of this country affords. But it is clearly the interest of the College (we mean the mammon interest) to make the path to the diploma as smooth and easy as possible. It has no power to *drive*, and therefore it must *draw*. To expect then that the College of Surgeons will ever voluntarily institute a rigid public examination of candidates, is to expect in a corporate body the complete victory of philanthropy over self-interest. On these arguments and facts we found our proposition that the surgical curriculum and the examination should be chalked out by a power higher than that of the College, and free from all pecuniary interest in the effects of the curriculum and the examination.

It has been said of men, that some are born to honours—some acquire honours, and others have honours thrust upon them. In respect to the Apothecaries' College, it certainly has had honours—or at least powers, thrust upon it. It is not the least anomaly in this chaos of anomalies, that the lowest person in the medical trinity should be invested with the greatest trust. The College of Physicians cannot prevent a quack from practising as a physician—the College of Surgeons cannot prevent the veriest Charlatan from undertaking the most important surgical operations—but the Apothecaries' Company can prevent Hippocrates, Celsus, or Sydenham, if they were to rise from their graves, cloathed in flesh and blood, and endowed with all their pristine knowledge, from practising as a surgeon-apothecary, unless they submitted themselves for examination in the laboratory of drugs, oils, and colours, and bought the necessary admeasurement of the PATENT! A man may study and graduate in Paris, Vienna, Edinburgh, Dublin, or Glasgow—he may spend twenty years in acquiring all kinds of medical, surgical, and pharmaceutical knowledge—and still he dare not sit down in the remotest nook of this land of liberty, to practise as a general practitioner, without paying the tax in Apothecaries' Hall, or subjecting himself to a law-suit! Now, as the Worshipful Company, like the other two Companies, are annually chopping and changing their little CURRICULA, why is it that the said Worshipful Company does not encourage the acquirement of medical knowledge and medical honours, by exempting the graduates of regular Universities, who choose to practise in the same way as did HIPPOCRATES, CELSUS, GALEN, MORGAGNI, and the greatest men of their respective ages (who were all general practitioners) from the degrading tax of undergoing a mock-ceremony in Apothecaries' Hall? The reason is obvious. The motto in Union-street, is the same as in Lincoln's-Inn Fields and Pall-mall East—VIRTUS *post* NUMMOS!

Without descending, in the present paper, to a minute detail of the evils and absurdities which abound in the three heads of departments, and which have thence spread through every ramification of the profession, we may fearlessly assert that this outline (which we maintain to be correct) will convince every unprejudiced man, that nothing but a complete reconstruction of the whole system of medical polity, in this country, can offer any hope of success. It is quite needless to expect that the corporate bodies will rectify these laws, unless human nature can be changed, and the love of their neighbours made to predominate over the love of themselves. What are the remedies then for this tremendous mass of evils? Certainly they are not to be sought in scurrilous personal abuse lavished on individual members of these corporations—nor on insulated petitions from single departments of the profession to the legislature. All classes of medical society suffer from these evils—then why should not all unite to obtain redress? The great object would be, to calmly but forcibly point out the distracted state of the profession, and the discordancy as well as the absurdity of existing regulations—and pray for a parliamentary enquiry, without proposing any schemes of reform at all. It should be left to the wisdom of a parliamentary committee to call before them men from all ranks of the profession, and thereby get at the sentiments of medical men in the aggregate. Such a petition should breathe no sentiments that might militate against the success of the great cause. It should call for no thunderbolts to be hurled down on this or that edifice—no eradication of this or that charter. It should propose no visionary schemes—but merely point out the evils, and leave it to our legislators to find the remedy. We conceive that the various ills by which we are now oppressed, might be removed without annihilating a single chartered body, or disturbing a single grade or distinction which now exists in the profession. If one salutary code of laws were established by the legislature, the existing corporate bodies might, with certain precautions, be entrusted with the administration of them. The evil is, that there is no general system of medical education, and each corporate body issues, from time to time, its little narrow-minded regulations, producing incessant turmoil and confusion in practice as well as study. If a minimum of medical study were established, below which it would be illegal to practise—it might fairly be left to each individual's choice to go as far beyond that boundary, upwards, as he pleased. Thus, for example, suppose three, or any other number of years' regular study, and then a public examination, were enjoined—the curriculum being fixed by Government, and not left to the caprice of any corporate body?—then it should be lawful for the student to present himself at any one, two, or three of the colleges, for examination, and load himself with as many honours and diplomas as his intellects, his acquirements, and his ambition aspired to. The public examination over, he should be left to practise any department of the profession he pleased, without let or hindrance. But, as medical science is indivisible in its principles and elements—the same course of study should be prescribed for all—and also the same kind of public examination. Let the *minimum* of education embrace a fair range of the science—and let the maximum be without bounds, for all those whose zeal, talents, or means, enabled them to soar beyond the ordinary limits. But we repeat it, that all these regulations should emanate from the legislature, and not a single suggestion should appear on the face of the petition. The catalogue of evils is quite long enough for any prayer to parliament—let redress flow from the proper source.

It only remains to advert to the steps which are necessary to be taken. Hercules will not help the waggon out of the slough, unless we put our shoulders to the wheels. If all sit quiescent, and each leaves it to his neighbour to move first, all hopes of reformation are gone. The impulse should be simultaneous through the three classes of the profession. All distinctions should be thrown aside—and all should unite in framing a petition to the legislature. A precipitate and intemperate appeal from any one branch, will endanger the whole cause. A public meeting should be called in the course of the Summer, and as many of our country brethren as possible should attend. The resolutions might then be entered into, and a committee, composed of physicians, surgeons, and general practitioners, should be appointed, to draw up a temperate but energetic delineation of the state of the profession, for circulation among the members of parliament, with the view of paving the way for a formal petition to the legislature itself. This, in our opinion, is the only plan that offers any prospect of final success. If they make no effort, they deserve to remain for ever in a state of thraldrom and degradation.

